# Monitoring the Reactivity of Formamide on Amorphous SiO_2_ by In-Situ UV-Raman Spectroscopy and DFT Modeling

**DOI:** 10.3390/molecules25102274

**Published:** 2020-05-12

**Authors:** Matteo Signorile, Stefano Pantaleone, Nadia Balucani, Francesca Bonino, Gianmario Martra, Piero Ugliengo

**Affiliations:** 1 Dipartimento di Chimica and Nanostructured Interfaces and Surfaces (NIS) Centre, Università degli Studi di Torino, via P. Giuria 7, IT-10125 Torino, Italy; matteo.signorile@unito.it (M.S.); stefano.pantaleone@univ-grenoble-alpes.fr (S.P.); francesca.bonino@unito.it (F.B.); gianmario.martra@unito.it (G.M.); 2 Dipartimento di Chimica, Biologia e Biotecnologie, Università degli Studi di Perugia, Via Elce di Sotto 8, I-06123 Perugia, Italy; nadia.balucani@unipg.it

**Keywords:** formamide, nucleobases, silica, UV-Raman, DFT calculations

## Abstract

Formamide has been recognized in the literature as a key species in the formation of the complex molecules of life, such as nucleobases. Furthermore, several studies reported the impact of mineral phases as catalysts for its decomposition/polymerization processes, increasing the conversion and also favoring the formation of specific products. Despite the progresses in the field, in situ studies on these mineral-catalyzed processes are missing. In this work, we present an in situ UV-Raman characterization of the chemical evolution of formamide over amorphous SiO_2_ samples, selected as a prototype of silicate minerals. The experiments were carried out after reaction of formamide at 160 °C on amorphous SiO_2_ (Aerosil OX50) either pristine or pre-calcined at 450 °C, to remove a large fraction of surface silanol groups. Our measurements, interpreted on the basis of density functional B3LYP-D3 calculations, allow to assign the spectra bands in terms of specific complex organic molecules, namely, diaminomaleonitrile (DAMN), 5-aminoimidazole (AI), and purine, showing the role of the mineral surface on the formation of relevant prebiotic molecules.

## 1. Introduction

**Figure 1:**
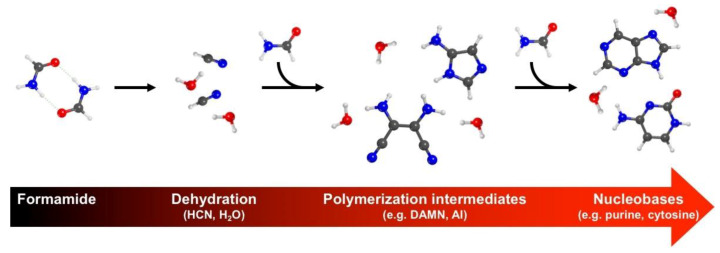
Schematic reaction path from formamide to form nucleobases, via its dehydration to HCN and H_2_O and its subsequent polymerization reaction through stable intermediates (examples represented: diaminomaleonitrile, DAMN; and 5-aminoimidazole, AI) to yield nucleobases (examples represented: purine and cytosine). Atoms color code: white, H; gray, C; blue, N; and red, O.

The understanding of atomistic processes underlying the origin of life is nowadays a hot multidisciplinary research topic [1]. From the chemical point of view, the focus is on the elucidation of mechanisms leading from isolated atoms in a rarefied cosmic environment to the biomolecular complexity characterizing living creatures. Discoveries from astrochemical observations in some specific space regions (e.g., dense clouds or cometary comae) have revealed that relatively complex molecules are present [2]. Among them, a very interesting one is formamide [2], since: i) it is the simplest molecule containing an amide bond, present in many fundamental biopolymers (e.g., peptides); ii) it contains four over six of the fundamental elements of biomolecules; and iii) it is relatively stable, but it can decompose into very reactive species, in particular HCN, via dehydration [3,4,5,6,7]. The last point is of particular interest with respect to the formation of the building blocks of biopolymers: HCN polymerization can easily lead to nucleobases as originally discovered by Orò for adenine [8], i.e., the main constituents of nucleic acids, also involving stable intermediate compounds [6,9,10,11,12]. This concept is summarized in Figure 1. This reactions cascade can take place in bulk formamide in presence of an energy source [6,9,10,13], however it has been demonstrated to be significantly boosted in presence of mineral phases in the reaction environment [13,14,15,16,17,18,19,20]. Such catalytic effect does not only affect the nucleobases productivity, but also causes its selective conversion toward specific products depending on the nature of the mineral component. In this way, the five fundamental nitrogenous bases (adenine, guanine, cytosine, thymine and uracil) can be generated from the sole formamide by exploiting the different selectivity provided by specific mineral catalysts [13,14,15,16,17,18,19].

Among the plethora of relevant inorganic phases that could act as catalysts, silicates certainly assume a prominent role due to their abundance. Indeed, silicates are a broad family of minerals, being the most common constituents of the Earth’s crust [21]. Nonetheless, they are also found in astronomical environments, e.g., the core of dust grains in nebulae and interstellar regions [22]. The reactivity of several silicates has been tested vs. formamide decomposition, and even the simplest materials (quartz and silica) demonstrated their ability in driving the reaction toward defined products [5]. In this work, we deal with the study of the formamide reactivity in presence of pyrogenic silica samples with different surface silanols population. The adoption of an amorphous silica rather than a crystalline one (which is more relevant in a prebiotic context) is due to the favorable properties of this type of material, providing much higher specific surface area with identical composition and similar types of surface terminations (i.e., silanols patches characterized by different intersilanols interactions [23]), making it a more convenient sample for spectroscopic/catalytic studies. We monitored the reaction of formamide by in situ UV-Raman spectroscopy: the advantage of this technique is the capability of exploiting the electronic transition in the UV portion of the spectrum, characterizing many organic molecules and inorganic moieties (as well as nucleobases, see Figure S1), in order to achieve resonance conditions. In this way, the intensity of specific vibrational modes (principally those having the same symmetry of the involved electronic transition) is selectively enhanced by orders of magnitude. Accordingly, resonant UV-Raman could become very sensitive to highly diluted species, such as metal dopants in non-absorbing matrices [24] or adsorbed molecules [25]. This increased sensitivity also makes resonant Raman suitable for time resolved studies, e.g., the in situ characterization of reactions happening at a catalyst surface [26,27,28,29]. By adopting resonant UV-Raman, we present here unprecedented insights on the effect of surface features of model mineral phases (i.e., amorphous silica samples with different surface silanols population) toward the conversion of formamide to nucleobases. The interpretation of the complex datasets is supported by quantum mechanical simulations based on density functional theory, helping to access the spectroscopic features of molecular species otherwise hardly identifiable from the sole experiment (e.g., stable reaction intermediates).

## 2. Results

**Figure 2:**
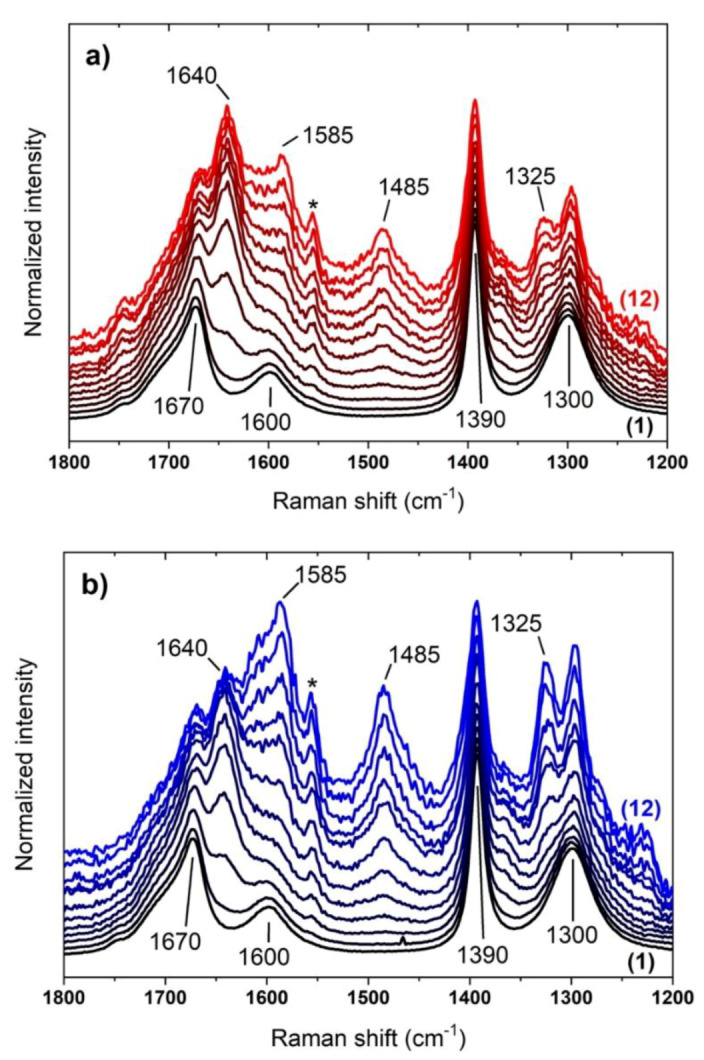
UV-Raman (λ= 266 nm) spectra collected in situ during 12 h of reaction of formamide at 160 °C on amorphous SiO_2_ (Aerosil OX50): (**a**) as such; and (**b**) calcined at 450 °C. A spectrum per hour was collected (time evolution from (1) black to (12) red/blue), in order to obtain a sufficient signal-to-noise ratio. Spectra have been normalized to the intense feature of formamide at 1390 cm^−1^ and vertically shifted for the sake of visualization. The asterisk (*) marks the feature associated to atmospheric O_2_, as present in the optical path of the instrument.

In Figure 2, the UV-Raman spectra of the formamide-silica suspension collected along 12 h of reaction at 160 °C are shown.

During the early reaction stages (up to 2 h), the spectral evolution is negligible regardless of the SiO_2_ sample used, and the main peaks closely match the Raman spectrum of pure formamide in liquid phase [9]. The four prominent signals observed can be assigned to the symmetric and antisymmetric stretching of the amide moiety (1300 and 1670 cm^−1^, respectively), to the in-plane NH_2_ bending (1600 cm^−1^) and to the in-plane CH bending (1390 cm^−1^). The extended tail toward higher wavenumber characterizing the antisymmetric amide stretching suggests that the formamide molecules are interacting with different species, most probably other formamide molecules or, eventually, silanol groups of the SiO_2_ surface (in agreement with previous report [30]). In the following hours, several spectral modifications occur, in particular the formation of new bands is observed. The most relevant ones are centered at 1325, 1485, 1585 and 1640 cm^−1^. The last peak can be tentatively assigned to water, specifically to its bending mode, produced as a consequence of the formamide dehydration process. The remaining bands are instead compatible with the Raman signature of nucleobases and related molecules [31], a reactivity occurring in a very limited extent when heating pure formamide [13]. Interestingly, these fingerprints are present for both the bare and the calcined silica samples, testifying as the nature of the products of formamide decomposition is very similar despite the difference in the surface properties of the two materials. Nevertheless, the relative intensity of bands due to nucleobases and water molecules appears dependent on the surface silanols population, providing a clear evidence of the key role of the silica surface in promoting the observed reactivity. Furthermore, in the case of the bare SiO_2_ an additional feature appears at ~1750 cm^−1^ at late reaction stages, likely monitoring the formation of a compound containing a carbonylic moiety [32]. A sharp peak at 1555 cm^−1^ also rises for both the samples along reaction time: this is due to the ν(O=O) stretching mode of atmospheric O_2_ as present in the optical path in the Raman spectrometer [33]. The apparent increase in intensity during time of this component, despite the constant length of the beam path, results from the combined effect of the progressive decay of the UV-Raman spectra overall intensity, due to the formation of self-absorbing species, and the adopted data normalization process.

**Figure 3:**
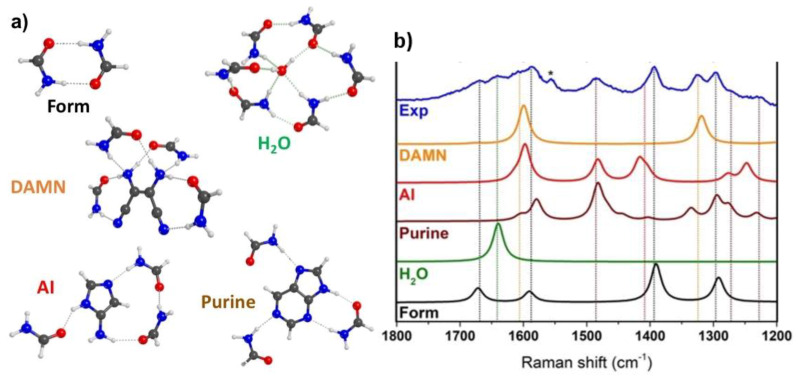
(**a**) Optimized B3LYP-D3/TZVP structures of the formamide micro-solvated species: Form (formamide), water (H_2_O), diaminomaleonitrile (DAMN), 5-aminoimidazole (AI) and Purine (Purine). (**b**) Experimental UV-Raman (λ= 266 nm) spectrum of formamide at 160 °C on amorphous SiO_2_ (Aerosil OX50, calcined at 450 °C) after 12 h of reaction (Exp, blue curve) compared to the B3LYP-D3/TZVP anharmonic Raman spectra for DAMN (orange curve), AI (red curve), Purine (brown curve), H_2_O (green curve) and Form (black curve). The asterisk (*) marks the feature associated to atmospheric O_2_, as present in the optical path of the instrument.

Except for the 1640 cm^−1^ peak, ascribed to water, the other features in the experimental UV-Raman spectra collected during formamide reaction are not straightforwardly assignable to specific molecules. In order to assess their origin, we relied on B3LYP-D3/TZVP based calculations to simulate Raman spectra of some potentially relevant products/intermediates. The adopted computational level was carefully selected among other possibilities by comparing the computed Raman spectrum of liquid formamide with the experimental one (see the Supplementary Material for further details). According to previous literature, the principal product during formamide decomposition over silica is purine [13], which has been proposed to be formed via HCN polymerization, also involving stable intermediates such as diaminomaleonitrile (DAMN) and 5-aminoimidazole (AI) [9,10,11]. To mimic the formamide environment in which these molecules may be formed, we solvated each species of interest with the minimum amount of formamide molecules (micro solvation) and embedded the system in a conductor-like polarized continuum model (C-PCM). On the fully optimized structures of the solvated species we computed the Raman spectrum for the interested species taking anharmonicity into account through perturbation theory and evaluating the static Raman intensities. These results are reported in Figure 3.

With the help of simulated spectra, we can easily recognize the formation of DAMN, as indicated by the growth of its principal Raman fingerprints at 1325 and 1610 cm^−1^. We also clearly identify purine, from its most intense feature at 1485 cm^−1^. However, other relevant peaks could be present and overlapped to the intense signals of formamide, justifying the change in their relative intensities (e.g., the intensity ratio of the 1390 over the 1300 cm^−1^ band passes from 0.54 at 1 h of reaction to 0.85 after 12 h). Additionally, a weak signal can be distinguished from background at around 1230 cm^−1^ and a shoulder is present at 1270 cm^−1^, both ascribable to purine. Finally, since several of its spectral features overlap with both DAMN and purine, we cannot exclude/confirm the formation of AI, possibly identified by a high frequency shoulder of 1390 cm^−1^ band of formamide. For the sake of completeness, we also simulated the spectrum of water solvated by formamide: consistently with our assignment, the δ(HOH) bending mode is found at 1640 cm^−1^, confirming the formation of water in the reaction environment.

**Figure 4:**
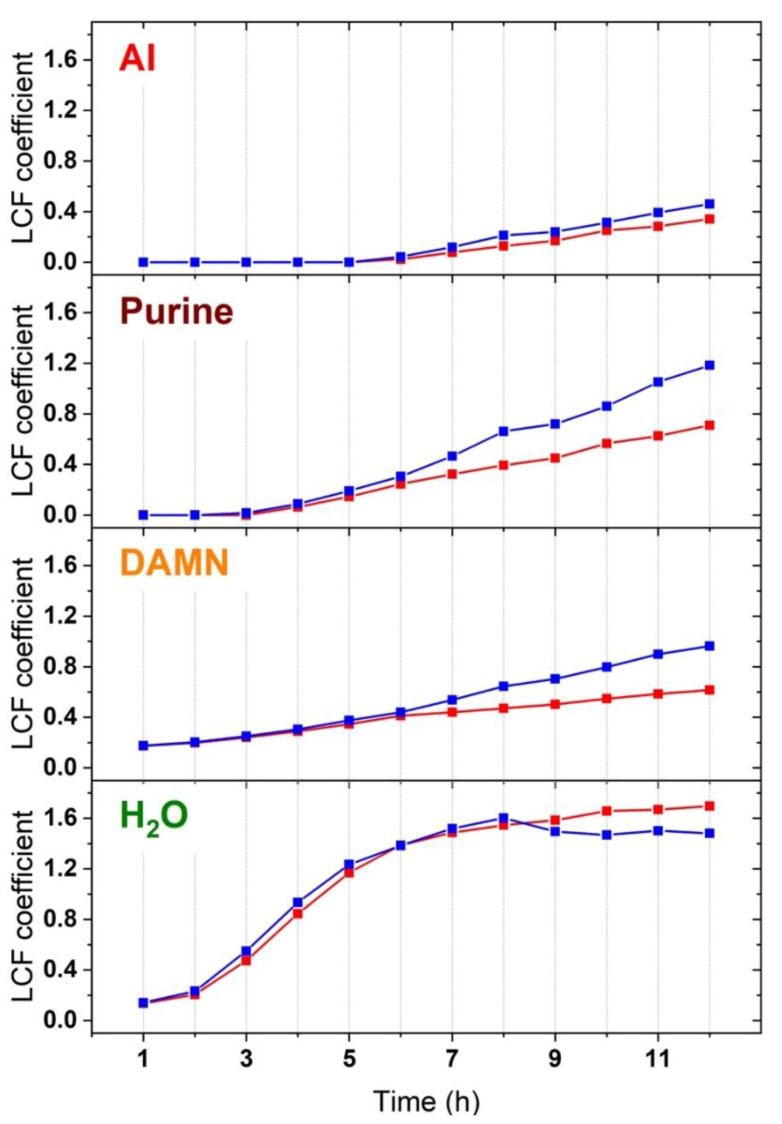
LCF coefficients vs. time of molecules formed during formamide reaction (H_2_O, DAMN, Purine and AI), derived from datasets reported in Figure 2, as measured during 12 h of reaction of formamide at 160 °C on amorphous SiO_2_ (Aerosil OX50): as such (red); and calcined at 450 °C (blue).

Being the main reaction components and their Raman spectra identified, we performed a linear combination fit (LCF) of the datasets presented in Figure 2 by using the five calculated spectra as base. An example of fitted experimental spectrum is presented in Figure S2. The time evolution for the obtained LCF coefficients of products/intermediates (formamide is omitted since set as constant, see the Materials and Methods section) are presented in Figure 4.

The LCF coefficients for each considered component show an increasing trend along the 12 h of reaction, testifying the progressive formation and accumulation of the related intermediates/products. H_2_O coefficients reach a plateau value after growing during the first 7 h of reaction, when they stabilize around a closely constant value along the last 5 h of reaction. Due to the complexity of the data treatment and to the overlap of several spectroscopic fingerprints in the vicinity of the 1640 cm^−1^ peak, the small variations observed for the H_2_O behavior between the two SiO_2_ samples cannot be safely correlated to a specific role of the catalyst. The other considered components (DAMN, AI and purine) present a monotonic increase of their coefficients along the whole experiment. In detail, DAMN is formed first (it is already detected at early reaction stages), and it constantly increases until 6 h of reaction for both samples, when its concentration rises faster in the case of the calcined SiO_2_. A similar trend is recognized for both purine and AI: the former becomes detectable since 3–4 h of reaction and has a significant increase after 6 h (in particular for the calcined sample). The latter is instead observed at late reaction stages, after 6–7 h, and the magnitude of its LCF coefficients is not significantly different between the two samples.

## 3. Discussion

The combination of UV-Raman data with DFT simulation allows describing the formation of nucleobases (in this specific case, purine) via dehydrative decomposition of formamide. Furthermore, we are also able to distinguish a different reactivity behavior of the system depending on the surface properties of the SiO_2_ mineral phase. In agreement with previous studies, we effectively detected the formation of purine as main reaction product [13]. However, the same study reports also cytosine should be produced with similar concentration. We can justify the impossibility to detect cytosine by considering the particular measurement conditions we adopted, i.e., resonant Raman. Despite all nucleobases have electronic transitions in the vicinity of the excitation wavelength, the absorption coefficients for purines are about twice as large as those of pyrimidines [34], justifying the stronger contribution of purine to the resonant Raman spectra (see also Figure S1). This situation is analogous to that observed during the methanol to hydrocarbons reaction, where the main products are olefins and methylated benzenes, but the resonant UV-Raman spectra are dominated by the signals from polycyclic aromatic hydrocarbons, much less abundant in the reaction environment but characterized by higher absorption coefficients [26]. Beside the final product, we also detected intermediate species on the basis of experimental and computational studies [6,9,10,11,12]. In detail, we assessed the formation of DAMN, a stable HCN tetramer recognized as a purine/adenine precursor. The AI formation is instead hardly detectable by direct data analysis; however, its production is confirmed via LCF, where AI concentration becomes nonzero at late reaction time.

Another relevant insight concerns the time evolution of the process which can be described as a function of the surface properties of the mineral phase (here amorphous SiO_2_) acting as a catalyst. The concomitance of the stabilization of the amount of H_2_O in the suspension and the increase in the product/intermediates concentration suggests the process initially mostly involves the dehydration of formamide to HCN and H_2_O, as testified by the increase in concentration of H_2_O along the early reaction stages. Recent computational studies highlighted as this process is favored in aqueous solution [7], as well as on silica surfaces [5], in comparison to bare formamide in gas phase. Also high-energy conditions (e.g., under irradiation or at elevated temperatures [6]) have been demonstrated to favor the formamide decomposition to cyanides and their polymerization to yield nucleobases. Our findings, discussed hereafter, confirm this scenario. The formed HCN readily reacts to give the stable DAMN, already detectable at the beginning of the process. After 7 h, the concentration of H_2_O in the suspension stabilizes, suggesting a steady-state conversion is reached in the dehydration step. This equilibrium conversion could be the consequence of the accumulation of purine, displacing the reaction toward reagents and causing the stabilization of the formamide dehydration step. As a consequence of this, also the concentration of stable reaction intermediates (DAMN, AI) increases, after H_2_O reaches the plateau. Interestingly, the magnitude in the rise of the concentration of product/intermediates is larger in the case of the calcined SiO_2_ compared to the bare material. We can ascribe the behavior in the two materials to their different population of surface species (i.e., silanol groups). As presented in Figure S3 of the Supplementary Material, the bare Aerosil OX50 presents a significant fraction of silanols mutually interacting through H-bonds beside isolated/weakly interacting silanols, whereas the latter are the dominant surface species after calcination. Since the dehydration stage occurs with a similar reaction rate on both catalysts (in agreement with our previous computational investigation [5]), we can infer the HCN polymerization at the SiO_2_ surface is favored on the isolated/weakly interacting silanols. Interestingly, the different surface sites on the two studied silica samples also slightly affect the products selectivity, since minor features (i.e., the weak signal at ~1750 cm^−1^ observed for the as such SiO_2_, tentatively assigned to a carbonyl-containing molecule [32]) are exclusively observed for bare SiO_2_. This alternative reactivity could be due to the presence of the interacting silanol at the catalyst surface, promoting a different reaction path.

In conclusion, this work proves the effectiveness of combining computer modeling based on DFT calculations with resonant UV-Raman, as a selective probe to identify reaction intermediates/products in the formamide conversion to nucleobases in solvothermal conditions. The interplay between the two approaches is essential to assign the spectral features of an otherwise complex set of experimental spectra. With respect to previous studies, the present one also provides unprecedented insights on the effect of surface properties of silica catalysts on the formamide conversion to nucleobases, thus helping in the elucidation of the role of a mineral phase in the reaction environment. The same method will be applied in the future to a broad set of mineral phases, in order to disclose their role in the origin of molecules of life.

## 4. Materials and Methods

### 4.1. Materials

Formamide (spectrophotometric grade, ≥99%), Aldrich, Saint Louis, MO, USA) was used as received, without further purification. Aerosil OX50 pyrolitic silica was supplied by Evonik (Essen, Germany). The sample was used as such or after calcining it, according to the procedure reported by Rimola et al. [35]. In brief, silica was pelletized with a press and heated in static air up to 450 °C (30 min ramp) for 2 h, then it was cooled back to r.t. in the closed furnace. The effect of the calcination is to reduce irreversibly (by subsequent contact with H_2_O vapour at 20 mbar) and in a controlled way the population of surface silanols, in terms of a depletion of H-bonded silanols, in favor of isolated/weakly interacting silanols. The IR spectra of the two samples in the OH stretching region are presented in Figure S3 of the Supplementary Material and commented herein.

### 4.2. Experimental Methods

UV-Raman spectra were collected at the BL10.2-IUVS beamline at the Elettra synchrotron (Trieste, Italy) [36], at an excitation wavelength of 266 nm. The light backscattered by the sample was filtered by an edge filter (removing the contribution from Rayleigh scattering), analyzed by a Trivista 557 spectrometer (1800 lines/mm grating, Princeton Instruments, Trenton, NJ, USA) and detected by a Peltier cooled UV-enhanced CCD.

In a typical experiment, 30 mg of silica were loaded in a UV-grade cuvette and contacted with 2.65 mL of formamide (1:100 SiO_2_:formamide weight ratio). SiO_2_ was suspended in formamide by continuous magnetic stirring. The reaction mixture was rapidly heated to 160 °C by a resistive element, then spectra collection began: 12 h of reaction were monitored. The adopted reaction conditions are based on those reported by Saladino and coworkers on the same material [13]. The UV-Raman spectra were collected by sampling the formamide-silica dispersion. Since the high flux of energetic photons irradiating the sample for a long time framework is known to potentially cause the undesired photoinduced decomposition of reactants/products [37], the cuvette was kept in a continuous oscillatory movement under the excitation beam. The spectra here reported are normalized to the intense feature of formamide at 1390 cm^−1^, assuming its concentration is constant over the reaction time (in agreement with the low conversion to nucleobases reported in the literature [13]). This procedure was necessary in order to compensate the stochastic fluctuations due to instrumental reasons, as well as the progressive drop in overall intensity associated to the formation of self-absorbing species.

### 4.3. Computational Methods

The aim of calculations here reported is to improve the assignment of the experimental UV-Raman spectral features, by simulating the vibrational frequencies and the associated Raman intensities for a selected pool of reaction products [13] and intermediates [9,10,12] in formamide solution. The cluster calculation included in this work were performed with the Gaussian16 (rev. B.01) code [38], adopting the B3LYP functional [39,40]. All the atoms were described through the Ahlrichs TZVP basis set [41], which was chosen according to its intrinsically small basis set superposition error. Dispersive forces were included through the D3 empirical scheme proposed by Grimme [42], using the Becke-Johnson damping scheme [43]. In order to properly describe solvation, we proceeded as follows: (i) we included explicit formamide molecules in direct interaction with the molecule of interest (typically interacting through hydrogen bond); and (ii) we run all the calculation including the conductor-like polarized continuum model (C-PCM) [44,45] with the dielectric constant of formamide (ε = 109.0). The structures of the initial models were fully optimized (including explicit solvent molecules), whereas frequency calculations were performed on the product/intermediate atoms only (keeping the solvent molecules frozen). This choice was mandatory, as frequency calculations were performed including the computational demanding anharmonic corrections and static Raman intensities [46,47,48], as the majority of vibrations includes hydrogen atoms, for which the anharmonic behavior is not negligible. When possible, symmetry was exploited to speed up the calculations, always taking care that all frequencies were real (otherwise symmetry was lowered or switched-off completely). Each part of the computational setup (method, basis set, implicit and explicit solvation models) was carefully calibrated on the formamide spectrum, as shown in the Supplementary Material (Section S4, Figures S4–S7). The obtained vibrational frequencies and Raman intensities were convoluted to build up the simulated spectra by adopting Lorentzian functions (FWHM = 10 cm^−1^). An example of Gaussian input for the Raman spectra simulation is provided in Section S5 of Supplementary Material. The optimized atomic coordinates for the five models adopted in the calculations are given as well in Section S6.

### 4.4. Linear Combination Fit (LCF)

The LCF procedure was applied to the experimental datasets, in order to describe them through the set of spectra derived from DFT calculations. In this way, a set of weight coefficients, directly proportional to the concentration of the species represented by each of the base spectra, was obtained. In detail, a least square algorithm was applied to each spectrum in order to minimize the following quantity:

$$\underset{C}{\min}||P\cdot C-D{||}_{2}^{2}$$

where *P* is a m × n matrix containing the pure spectra from DFT, *C* is a n × 1 vector containing the weight coefficients and *D* is a m × 1 vector containing the experimental data. The fit has been performed with the MATLAB^®^ software, imposing the weight coefficients to stay positive. Furthermore, the coefficient for the formamide component was fixed to 1. These constrains are physically justified, since: (i) the coefficients represent concentrations, which are positively defined; and (ii) the formamide concentration is constant along the whole experiment, since the conversion is low and it is present in large excess.

## Supplementary Materials

The following are available online. Figure S1. UV-vis spectra of cytosine and purine in formamide solution. Figure S2. Example of LCF. Figure S3. IR spectra of Aerosil OX50 samples in the OH stretching region. Figure S4. Simulated Raman spectra of formamide with different structural models. Figure S5. Simulated Raman spectra of formamide with different basis sets. Figure S6. Simulated Raman spectra of formamide with different methods. Figure S7. Simulated Raman spectra of formamide with different implicit solvation models.
