# Supplementary material for: Monitoring the Reactivity of Formamide on Amorphous SiO2 by In-Situ UV-Raman Spectroscopy and DFT Modeling
Source: Molecules. 2020 May 12;25(10):2274. doi: 10.3390/molecules25102274 (PMC7287731; doi:10.3390/molecules25102274)
Supplement: Supplementary file 1 [file molecules-25-02274-s001.pdf]

Supporting information for

Monitoring the reactivity of formamide on amorphous SiO_2 by in situ UV-Raman spectroscopy and DFT modeling

Matteo Signorile[1], Stefano Pantaleone[1,2], Nadia Balucani[2], Francesca Bonino[1], Gianmario Martra[1] and Piero Ugliengo[1,*]

[1] Dipartimento di Chimica and Nanostructured Interfaces and Surfaces (NIS) Centre, Università degli Studi di Torino, via P. Giuria 7, IT-10125, Torino, Italy;
[2] Dipartimento di Chimica and Nanostructured Interfaces and Surfaces (NIS) Centre, Università degli Studi di Torino, via P. Giuria 7, IT-10125, Torino, Italy;
* Correspondence: piero.ugliengo@unito.it;

S1. UV-vis spectra of cytosine and purine in formamide

Figure S1 shows the UV-vis spectra of cytosine and purine in formamide.

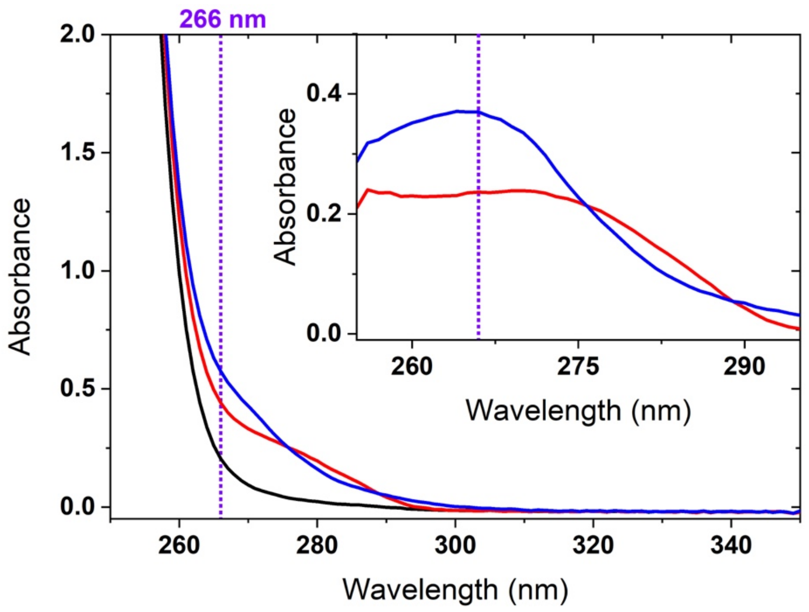

Figure S1. UV-vis spectra of: pure formamide (black curve); cytosine 10^{-4} M in formamide (red curve); and purine 10^{-4} M in formamide (blue curve). In the inset, the spectra of cytosine and purine are showed upon subtracting the solvent (i.e. formamide) contribution. The dotted violet vertical lines highlight the excitation wavelength adopted in the UV-Raman measurements.

The UV-vis spectra of cytosine and purine were collected as representative for the pyrimidines and purines nucleobases classes, also in agreement with their superior productivity in presence of silica as reported by Saladino et al. [1]. The typical electronic features of the analyzed nucleobases are observed as shoulders of the intense absorption of the solvent (i.e. formamide). Purine presents an higher absorbance with respect to cytosine at equal concentration (closely doubled according to solvent-subtracted spectra), in agreement with its larger absorption coefficient [2]. Also, the maximum of absorption of purine is closer to the excitation wavelength than that of cytosine.
The UV-vis spectra have been collected in transmission mode on a Varian Cary 300 spectrophotometer, within UV-grade quartz cuvettes (5 mm optical path).

S2. Example of linear combination fit (LCF)

Figure S2 shows an example of the LCF procedure outcome, here applied to the last spectrum of the calcined SiO_2 sample dataset.

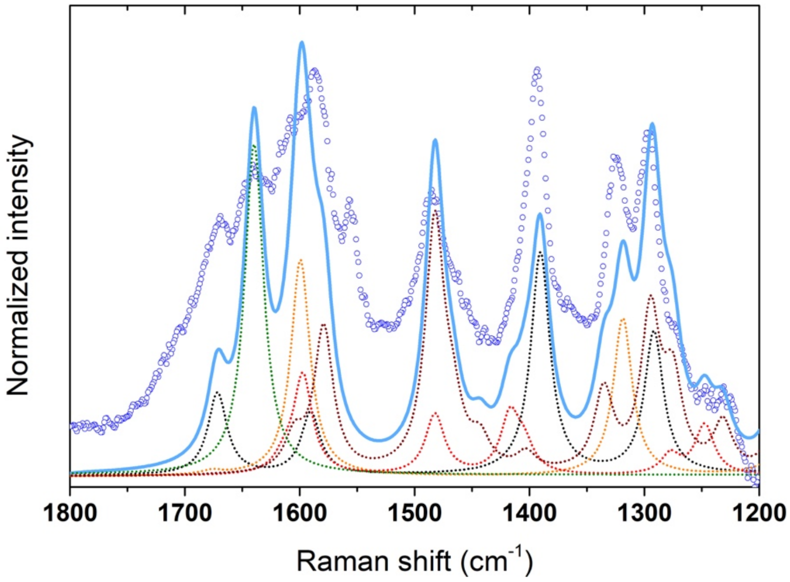

Figure S2. LCF procedure applied to the last spectrum of the calcined SiO_2 sample dataset (blue empty dots). The fitted spectrum is reported as solid cyan line. The coefficient-weighted base spectra are reported as dotted lines (DAMN, orange; AI, red; Purine, brown; H_2O, green; formamide; black).

The LCF procedure has been applied to the entire dataset presented in Figure 2 for both samples. The derived weight coefficients are presented in Figure 4 as a function of the reaction time. The LCF procedure is able to correctly reproduce the relative intensities of the main features observed in the experimental spectrum. However, due to the bandwidth set *a priori* to convolute the DFT-D3-computed spectra and not optimized during the fit, the spectral shape cannot be fully reproduced.

S3. IR spectra of Aerosil OX50, as such and calcined at 450 °C

Figure S3 shows the IR spectra of Aerosil OX50, as such and calcined at 450 °C, in the OH stretching region.

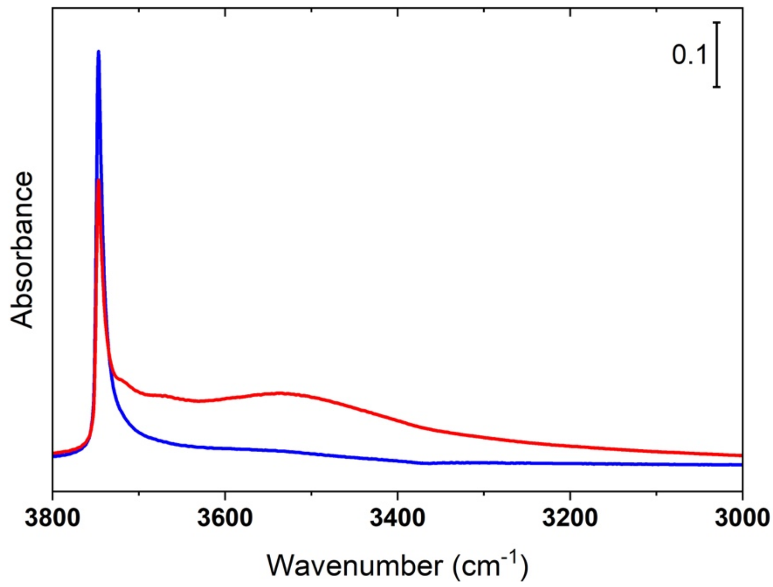

Figure S3. IR spectra of Aerosil OX50, as such (red curve) and calcined at 450 °C (blue curve), in the OH stretching region, collected after 30 min of outgassing at r.t.. The contribution of intraglobular silanols has been subtracted, according to the procedure described in ref. [3].

The IR spectra have been collected with a Bruker Vector 22 Fourier-transform spectrometer (DTGS detector), at a resolution of 4 cm^{-1} by averaging 100 scans. The samples, in form of self-supporting pellets, have been measured after 30 min of outgassing at r.t. in a home-made IR cell equipped with KBr windows. The contribution of intraglobular (i.e. internal silanols, not accessible by adsorbed molecules) has been subtracted by adopting the method proposed in the paper by Rimola et al. [3], based on the D_2O exchange of the external silanols. According to the literature [3], the calcination procedure causes a modification in the population of surface silanol groups. In detail, the silanols mutually interacting by H-bond (yielding the broad absorption in the 3200-3600 cm^{-1} range) decrease in number via irreversible condensation (towards subsequent adsorption of water vapour), leaving an increased number of weakly or not interacting silanols (absorbing in the 3730-3750 cm^{-1} range). Accordingly, the surface density of silanols decreases, causing an increased hydrophobicity for the calcined SiO_2 sample.

S4. Computational method optimization

The computational setup has been carefully tested on the formamide molecule in order to obtain a good match with its experimental measured Raman spectrum, both in terms of structural model and theoretical approach. The calibration of the structural model is shown in Figure S4, where the Raman spectrum of the single formamide molecule in the gas phase (red line) clearly does not match with the experimental spectrum (black line) of liquid formamide. At least the dimer of formamide (see Figure 3a) has to be included in the model (blue line), in order to account for the H-bond pattern present in liquid formamide, which strongly modifies the spectrum with respect to the single molecule. Here we remind that anharmonic corrections have been included in all the frequencies calculation performed to cope with the large anharmonicity of H containing bonds.

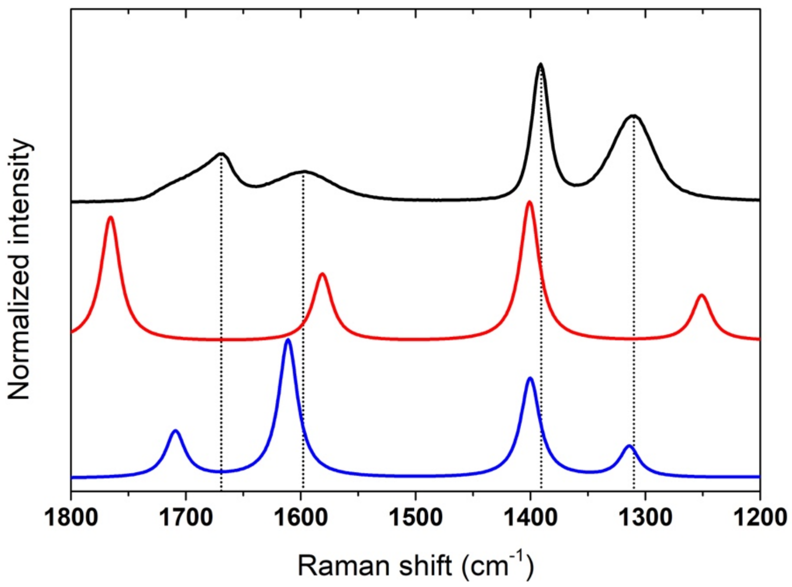

Figure S4. Simulated Raman spectra of formamide at B3LYP-D3/Ahlrichs TZVP level for different structural models: formamide monomer (red curve); and formamide dimer (blue curve). The experimental spectrum of liquid formamide (black curve) is reported for the sake of comparison.

Once the best model matching between experimental and simulated spectra has been obtained, we have calibrated the methodology to further improve the match. Figure S5 shows the calibration of the basis set, using the B3LYP-D3 method for all the calculations. Here the aim is to check the convergence of the basis set among the several calculations, rather than improving the match with the experimental spectrum. Results show that, apart the cheapest basis set used (6-31G(d,p)), all the others tested basis sets give similar results. Among them the Ahlrichs TZVP gives closely the same results of the cc-pVTZ, which is the most accurate and expensive basis set we tested here. Therefore, the Ahlrichs TZVP have been used for all other cases.

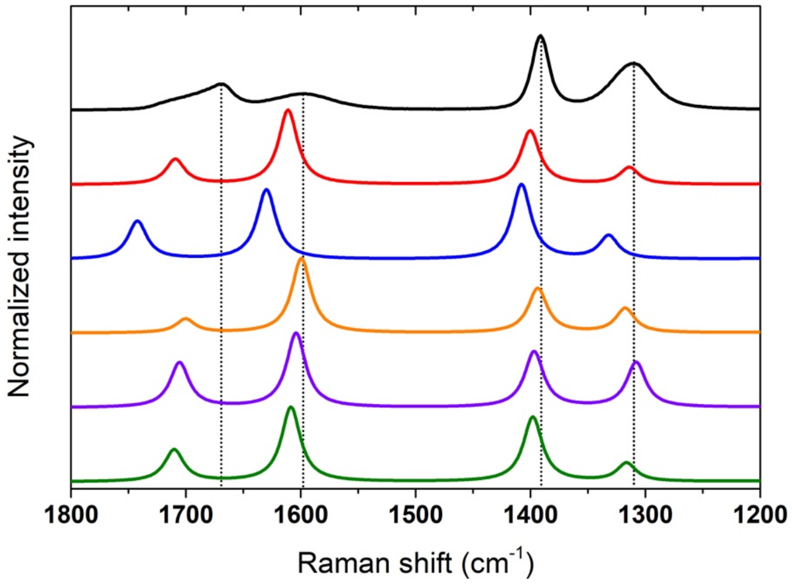

Figure S5. Simulated Raman spectra of formamide dimer at B3LYP-D3 level with different basis sets: Ahlrichs TZVP (red curve); 6-31G(d,p) (blue curve); 6-311+G(2d,2p) (orange curve); 6-311++G(3df,3pd) (violet curve); and cc-pVTZ (green curve). The experimental spectrum of liquid formamide (black curve) is reported for the sake of comparison.

The next step was the method calibration. Several density functional methods have been used (Figure S6), and it seems that the starting choice of the B3LYP-D3 (red curve) represents a good choice.

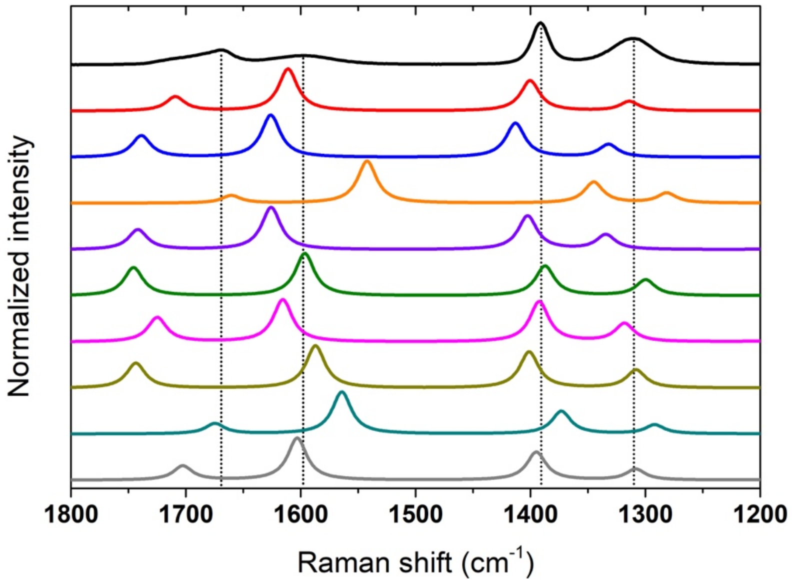

Figure S6. Simulated Raman spectra of formamide dimer with different methods and Ahlrichs TZVP basis set: B3LYP-D3 (red curve), CAMB3LYP-D3 (blue curve); PBE-D3 (orange curve); PBE0-D3 (violet curve); M06-D3 (green curve); M11 (magenta curve); MN15 (dark yellow curve); TPSS-D3 (dark cyan curve) and TPSSh-D3 (grey curve). The experimental spectrum of formamide (black curve) is reported for the sake of comparison.

Finally, as the experimental spectra have been recorded in liquid phase (formamide), the polarization continuum model has been included in the calculations (see Figure S7). Between the classical PCM and the conductor-like (C)-PCM, the latter seems to give better results.

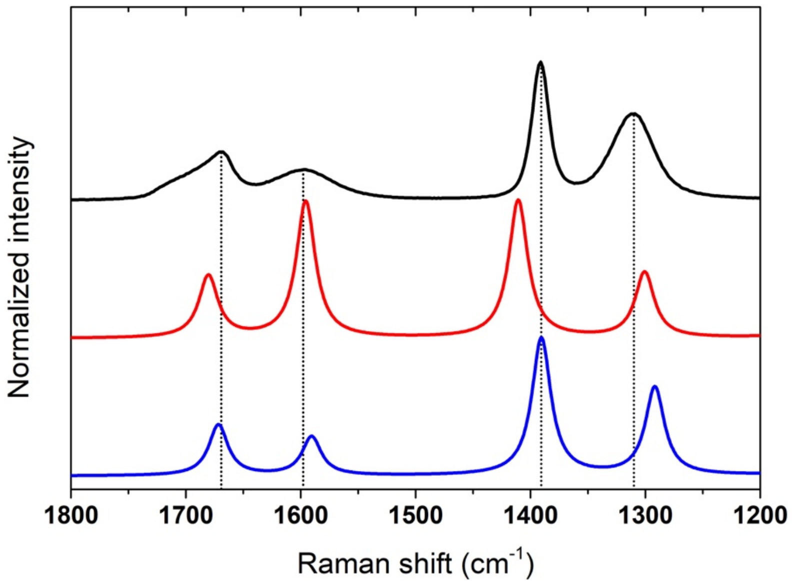

Figure S7. Simulated Raman spectra of formamide dimer at B3LYP-D3/Ahlrichs TZVP level with different implicit solvation models: PCM (red curve); and C-PCM (blue curve). The experimental spectrum of liquid formamide (black curve) is reported for the sake of comparison.

S5. Gaussian16 input file examples

In the following, two Gaussian16 input files used for geometry optimization and frequency calculation of purine are reported. For the sake of clarity, the atoms belonging to purine and to explicit solvent formamide molecules are specified. In the frequency calculation input file, the column in the middle between the atom types and the cartesian coordinates indicates the atoms free to move (labelled as 0) or frozen (labelled as -1), *i.e.* with no contribution in the spectrum simulation.

```
%nprocshared=16
%mem=64GB

#P B3LYP/gen opt(tight,maxcyc=100) int=superfinegrid scf(verytight) freq empiricaldispersion=gd3bj
   scrf(cpcm,solvent=formamide)

Optimization run

0 1
 6          1.107708     0.202912    -0.000000    #Purine
 6         -0.000000     1.076668     0.000000    #Purine
 6         -1.254754     0.488645     0.000000    #Purine
 6         -0.221753    -1.584088    -0.000000    #Purine
 1         -2.166222     1.075852     0.000000    #Purine
 1         -0.361928    -2.658708    -0.000000    #Purine
 1          3.191275     0.656667    -0.000000    #Purine
 7         -1.344707    -0.848304     0.000000    #Purine
 7          1.031946    -1.125460    -0.000000    #Purine
 7          0.447014     2.388494     0.000000    #Purine
 6          1.758099     2.292291     0.000000    #Purine
 7          2.214750     1.001837    -0.000000    #Purine
 1          2.437265     3.129733     0.000000    #Purine
 6         -2.918056     4.205752     0.000000    #Formamide
 1         -3.601683     5.069578     0.000000    #Formamide
 7         -1.625199     4.552472     0.000000    #Formamide
 1         -0.881041     3.847091     0.000000    #Formamide
 1         -1.373817     5.528137     0.000000    #Formamide
 8         -3.346687     3.053268     0.000000    #Formamide
 6          4.808268    -1.463599    -0.000000    #Formamide
 1          5.802784    -1.928338    -0.000000    #Formamide
 7          3.800737    -2.335078    -0.000000    #Formamide
 1          2.823059    -2.028370    -0.000000    #Formamide
 1          3.999655    -3.322875    -0.000000    #Formamide
 8          4.692416    -0.232430    -0.000000    #Formamide
 6         -3.192938    -4.059045    -0.000000    #Formamide
 1         -4.027421    -4.778219    -0.000000    #Formamide
 7         -3.590981    -2.780943     0.000000    #Formamide
 1         -2.903287    -2.017677     0.000000    #Formamide
 1         -4.574904    -2.564775     0.000000    #Formamide
 8         -2.022992    -4.437895    -0.000000    #Formamide

C    0
      S    5 1.
 0.8506038400000E+04   0.5337366400000E-03
 0.1275732900000E+04   0.4125023200000E-02
 0.2903118700000E+03   0.2117133700000E-01
 0.8205620000000E+02   0.8241786000000E-01
 0.2647964100000E+02   0.2401285800000E+00
      S    1 1.
 0.9241458500000E+01   0.1000000000000E+01
```

```
     S   1 1.
  0.3364353000000E+01   0.1000000000000E+01
     S   1 1.
  0.8717416400000E+00   0.1000000000000E+01
     S   1 1.
  0.3635235200000E+00   0.1000000000000E+01
     S   1 1.
  0.1287313500000E+00   0.1000000000000E+01
     P   4 1.
  0.3470949600000E+02   0.5330097400000E-02
  0.7959088300000E+01   0.3586581400000E-01
  0.2378697200000E+01   0.1420029900000E+00
  0.8154006500000E+00   0.3420310500000E+00
     P   1 1.
  0.2895378500000E+00   0.1000000000000E+01
     P   1 1.
  0.1008475400000E+00   0.1000000000000E+01
     D   1 1.
  0.8000000000000E+00   0.1000000000000E+01
****
N   0
     S   5 1.
  0.1191341675600E+05 -0.5229701736500E-03
  0.1786721383400E+04 -0.4042840403600E-02
  0.4065901283400E+03 -0.2077271512500E-01
  0.1149252506500E+03 -0.8118313784900E-01
  0.3710588342200E+02 -0.2387149739500E+00
     S   1 1.
  0.1297167619800E+02   0.1000000000000E+01
     S   1 1.
  0.4730229116400E+01   0.1000000000000E+01
     S   1 1.
  0.1252518425800E+01   0.1000000000000E+01
     S   1 1.
  0.5126007123100E+00   0.1000000000000E+01
     S   1 1.
  0.1793971400200E+00   0.1000000000000E+01
     P   4 1.
  0.4921875839200E+02   0.5552695347600E-02
  0.1134893530400E+02   0.3805461795700E-01
  0.3428508824600E+01   0.1494141224200E+00
  0.1179951255900E+01   0.3489818736800E+00
     P   1 1.
  0.4172612257700E+00   0.1000000000000E+01
     P   1 1.
  0.1429513128000E+00   0.1000000000000E+01
     D   1 1.
  0.1000000000000E+01   0.1000000000000E+01
****
O   0
     S   5 1.
  0.1590264745900E+05   0.5149980370300E-03
  0.2384953782900E+04   0.3981976442800E-02
  0.5427195718200E+03   0.2047697192200E-01
  0.1534040787400E+03   0.8026236791500E-01
  0.4954571614000E+02   0.2376683994700E+00
     S   1 1.
  0.1733964989700E+02   0.1000000000000E+01
```

```
      S    1 1.
 0.6330335527200E+01  0.1000000000000E+01
      S    1 1.
 0.1699588220100E+01  0.1000000000000E+01
      S    1 1.
 0.6895449127100E+00  0.1000000000000E+01
      S    1 1.
 0.2393602818100E+00  0.1000000000000E+01
      P    4 1.
 0.6327052401100E+02  0.6070920596000E-02
 0.1462331229500E+02  0.4194768872300E-01
 0.4448951800300E+01  0.1615688398800E+00
 0.1528151318000E+01  0.3568277929200E+00
      P    1 1.
 0.5299731587000E+00  0.1000000000000E+01
      P    1 1.
 0.1750944599800E+00  0.1000000000000E+01
      D    1 1.
 0.1200000000000E+01  0.1000000000000E+01
****
H  0
      S    3 1.
 0.3406134100000E+02  0.6025197800000E-02
 0.5123574600000E+01  0.4502109400000E-01
 0.1164662600000E+01  0.2018972600000E+00
      S    1 1.
 0.3272304100000E+00  0.1000000000000E+01
      S    1 1.
 0.1030724100000E+00  0.1000000000000E+01
      P    1 1.
 0.8000000000000E+00  0.1000000000000E+01
****


%nprocshared=16
%mem=64GB

#P B3LYP/gen int=superfinegrid scf(verytight) freq(raman,anharmonic) empiricaldispersion=gd3bj
 scrf(cpcm,solvent=formamide)

Anharmonic Raman frequency run

0 1
 6   0   1.107708   0.202912  -0.000000  #Purine
 6   0  -0.000000   1.076668   0.000000  #Purine
 6   0  -1.254754   0.488645   0.000000  #Purine
 6   0  -0.221753  -1.584088  -0.000000  #Purine
 1   0  -2.166222   1.075852   0.000000  #Purine
 1   0  -0.361928  -2.658708  -0.000000  #Purine
 1   0   3.191275   0.656667  -0.000000  #Purine
 7   0  -1.344707  -0.848304   0.000000  #Purine
 7   0   1.031946  -1.125460  -0.000000  #Purine
 7   0   0.447014   2.388494   0.000000  #Purine
 6   0   1.758099   2.292291   0.000000  #Purine
 7   0   2.214750   1.001837  -0.000000  #Purine
 1   0   2.437265   3.129733   0.000000  #Purine
 6  -1  -2.918056   4.205752   0.000000  #Formamide
 1  -1  -3.601683   5.069578   0.000000  #Formamide
```

7	-1	-1.625199	4.552472	0.000000	#Formamide
1	-1	-0.881041	3.847091	0.000000	#Formamide
1	-1	-1.373817	5.528137	0.000000	#Formamide
8	-1	-3.346687	3.053268	0.000000	#Formamide
6	-1	4.808268	-1.463599	-0.000000	#Formamide
1	-1	5.802784	-1.928338	-0.000000	#Formamide
7	-1	3.800737	-2.335078	-0.000000	#Formamide
1	-1	2.823059	-2.028370	-0.000000	#Formamide
1	-1	3.999655	-3.322875	-0.000000	#Formamide
8	-1	4.692416	-0.232430	-0.000000	#Formamide
6	-1	-3.192938	-4.059045	-0.000000	#Formamide
1	-1	-4.027421	-4.778219	-0.000000	#Formamide
7	-1	-3.590981	-2.780943	0.000000	#Formamide
1	-1	-2.903287	-2.017677	0.000000	#Formamide
1	-1	-4.574904	-2.564775	0.000000	#Formamide
8	-1	-2.022992	-4.437895	-0.000000	#Formamide

```
C    0
     S   5 1.
  0.8506038400000E+04   0.5337366400000E-03
  0.1275732900000E+04   0.4125023200000E-02
  0.2903118700000E+03   0.2117133700000E-01
  0.8205620000000E+02   0.8241786000000E-01
  0.2647964100000E+02   0.2401285800000E+00
     S   1 1.
  0.9241458500000E+01   0.1000000000000E+01
     S   1 1.
  0.3364353000000E+01   0.1000000000000E+01
     S   1 1.
  0.8717416400000E+00   0.1000000000000E+01
     S   1 1.
  0.3635235200000E+00   0.1000000000000E+01
     S   1 1.
  0.1287313500000E+00   0.1000000000000E+01
     P   4 1.
  0.3470949600000E+02   0.5330097400000E-02
  0.7959088300000E+01   0.3586581400000E-01
  0.2378697200000E+01   0.1420029900000E+00
  0.8154006500000E+00   0.3420310500000E+00
     P   1 1.
  0.2895378500000E+00   0.1000000000000E+01
     P   1 1.
  0.1008475400000E+00   0.1000000000000E+01
     D   1 1.
  0.8000000000000E+00   0.1000000000000E+01
****
N    0
     S   5 1.
  0.1191341675600E+05 -0.5229701736500E-03
  0.1786721383400E+04 -0.4042840403600E-02
  0.4065901283400E+03 -0.2077271512500E-01
  0.1149252506500E+03 -0.8118313784900E-01
  0.3710588342200E+02 -0.2387149739500E+00
     S   1 1.
  0.1297167619800E+02   0.1000000000000E+01
     S   1 1.
  0.4730229116400E+01   0.1000000000000E+01
     S   1 1.
```

```
   0.1252518425800E+01    0.1000000000000E+01
         S        1 1.
   0.5126007123100E+00    0.1000000000000E+01
         S        1 1.
   0.1793971400200E+00    0.1000000000000E+01
         P        4 1.
   0.4921875839200E+02    0.5552695347600E-02
   0.1134893530400E+02    0.3805461795700E-01
   0.3428508824600E+01    0.1494141224200E+00
   0.1179951255900E+01    0.3489818736800E+00
         P        1 1.
   0.4172612257700E+00    0.1000000000000E+01
         P        1 1.
   0.1429513128000E+00    0.1000000000000E+01
         D        1 1.
   0.1000000000000E+01    0.1000000000000E+01
****
O    0
         S        5 1.
   0.1590264745900E+05    0.5149980370300E-03
   0.2384953782900E+04    0.3981976442800E-02
   0.5427195718200E+03    0.2047697192200E-01
   0.1534040787400E+03    0.8026236791500E-01
   0.4954571614000E+02    0.2376683994700E+00
         S        1 1.
   0.1733964989700E+02    0.1000000000000E+01
         S        1 1.
   0.6330335527200E+01    0.1000000000000E+01
         S        1 1.
   0.1699588220100E+01    0.1000000000000E+01
         S        1 1.
   0.6895449127100E+00    0.1000000000000E+01
         S        1 1.
   0.2393602818100E+00    0.1000000000000E+01
         P        4 1.
   0.6327052401100E+02    0.6070920596000E-02
   0.1462331229500E+02    0.4194768872300E-01
   0.4448951800300E+01    0.1615688398800E+00
   0.1528151318000E+01    0.3568277929200E+00
         P        1 1.
   0.5299731587000E+00    0.1000000000000E+01
         P        1 1.
   0.1750944599800E+00    0.1000000000000E+01
         D        1 1.
   0.1200000000000E+01    0.1000000000000E+01
****
H    0
         S        3 1.
   0.3406134100000E+02    0.6025197800000E-02
   0.5123574600000E+01    0.4502109400000E-01
   0.1164662600000E+01    0.2018972600000E+00
         S        1 1.
   0.3272304100000E+00    0.1000000000000E+01
         S        1 1.
   0.1030724100000E+00    0.1000000000000E+01
         P        1 1.
   0.8000000000000E+00    0.1000000000000E+01
****
```

S6. Optimized atomic coordinates

In the following the optimized atomic coordinates discussed in the main text (*i.e.* Form, H_2O, Purine AI, DAMN) are reported in .xyz format.

```
12
Form
8    -1.139706    1.450750   -0.000000
7     1.139706    1.451308    0.000000
6    -0.056383    2.042351   -0.000000
1     1.975966    2.012959    0.000000
1     1.217404    0.429148    0.000000
1    -0.014315    3.140674   -0.000000
8     1.139706   -1.450750    0.000000
7    -1.139706   -1.451308   -0.000000
6     0.056383   -2.042351    0.000000
1    -1.975966   -2.012959   -0.000000
1    -1.217404   -0.429148   -0.000000
1     0.014315   -3.140674    0.000000
```

39

H_2O

8	-0.182371	0.014672	0.322346	#H_2O
1	-0.540921	0.005867	1.239189	#H_2O
1	0.506015	0.714852	0.268419	#H_2O
6	2.042463	2.842438	-0.544114	#Formamide
1	3.051648	3.262830	-0.632771	#Formamide
7	1.100354	3.552288	-1.147616	#Formamide
1	0.106316	3.296911	-1.149605	#Formamide
1	1.363045	4.397343	-1.630705	#Formamide
8	1.862765	1.787194	0.084193	#Formamide
6	-2.536277	-0.060885	3.104152	#Formamide
1	-2.839948	0.192855	4.128873	#Formamide
7	-3.556339	-0.396507	2.314640	#Formamide
1	-3.445236	-0.678292	1.339728	#Formamide
1	-4.489696	-0.398886	2.694811	#Formamide
8	-1.346666	-0.020866	2.777653	#Formamide
6	-3.010265	-2.214290	-0.936517	#Formamide
1	-3.413609	-3.123552	-1.401579	#Formamide
7	-1.688073	-2.122356	-0.963571	#Formamide
1	-1.221549	-1.316000	-0.553481	#Formamide
1	-1.077577	-2.843945	-1.344612	#Formamide
8	-3.769072	-1.366107	-0.440739	#Formamide
6	-2.629228	2.518536	-1.433169	#Formamide
1	-3.629354	2.750930	-1.825116	#Formamide
7	-2.510699	1.289487	-0.939538	#Formamide
1	-1.629330	0.966449	-0.538654	#Formamide
1	-3.281372	0.632659	-0.932337	#Formamide
8	-1.725245	3.359922	-1.470036	#Formamide
6	1.740323	-3.111145	-1.314085	#Formamide
1	2.695054	-3.593710	-1.570601	#Formamide
7	1.890615	-2.001612	-0.592098	#Formamide
1	1.094052	-1.449394	-0.281737	#Formamide
1	2.824844	-1.678834	-0.335044	#Formamide
8	0.669978	-3.601568	-1.689772	#Formamide
6	4.916744	-0.362912	1.034161	#Formamide
1	5.862213	-0.490502	1.580618	#Formamide
7	4.259598	0.754048	1.352082	#Formamide
1	3.385175	1.023225	0.892854	#Formamide
1	4.655923	1.384534	2.030991	#Formamide
8	4.542307	-1.204901	0.215215	#Formamide

31
Purine

6	1.107708	0.202912	-0.000000	#Purine
6	-0.000000	1.076668	0.000000	#Purine
6	-1.254754	0.488645	0.000000	#Purine
6	-0.221753	-1.584088	-0.000000	#Purine
1	-2.166222	1.075852	0.000000	#Purine
1	-0.361928	-2.658708	-0.000000	#Purine
1	3.191275	0.656667	-0.000000	#Purine
7	-1.344707	-0.848304	0.000000	#Purine
7	1.031946	-1.125460	-0.000000	#Purine
7	0.447014	2.388494	0.000000	#Purine
6	1.758099	2.292291	0.000000	#Purine
7	2.214750	1.001837	-0.000000	#Purine
1	2.437265	3.129733	0.000000	#Purine
6	-2.918056	4.205752	0.000000	#Formamide
1	-3.601683	5.069578	0.000000	#Formamide
7	-1.625199	4.552472	0.000000	#Formamide
1	-0.881041	3.847091	0.000000	#Formamide
1	-1.373817	5.528137	0.000000	#Formamide
8	-3.346687	3.053268	0.000000	#Formamide
6	4.808268	-1.463599	-0.000000	#Formamide
1	5.802784	-1.928338	-0.000000	#Formamide
7	3.800737	-2.335078	-0.000000	#Formamide
1	2.823059	-2.028370	-0.000000	#Formamide
1	3.999655	-3.322875	-0.000000	#Formamide
8	4.692416	-0.232430	-0.000000	#Formamide
6	-3.192938	-4.059045	-0.000000	#Formamide
1	-4.027421	-4.778219	-0.000000	#Formamide
7	-3.590981	-2.780943	0.000000	#Formamide
1	-2.903287	-2.017677	0.000000	#Formamide
1	-4.574904	-2.564775	0.000000	#Formamide
8	-2.022992	-4.437895	-0.000000	#Formamide

29

AI

6	-0.599005	-2.045004	-0.192060	#AI
7	0.645156	-1.760877	0.123277	#AI
6	0.635242	-0.471138	0.633628	#AI
6	-0.649365	0.019989	0.611672	#AI
7	-1.423462	-0.992566	0.095254	#AI
7	-1.194827	1.220971	1.043916	#AI
1	1.537051	0.020836	0.950430	#AI
1	-0.958344	-2.966290	-0.618623	#AI
1	-2.079736	1.437736	0.600105	#AI
1	-0.525999	1.981438	0.973397	#AI
1	-2.419708	-0.897323	-0.090106	#AI
6	4.344381	-1.397447	-0.045748	#Formamide
1	5.392081	-1.719528	-0.145204	#Formamide
7	3.463489	-2.386126	-0.201284	#Formamide
1	2.443166	-2.238556	-0.118825	#Formamide
1	3.805559	-3.316486	-0.383990	#Formamide
8	4.069838	-0.217750	0.191172	#Formamide
6	-5.106883	0.357753	-0.265968	#Formamide
1	-5.689438	1.286631	-0.212283	#Formamide
7	-5.856395	-0.738294	-0.412493	#Formamide
1	-5.427193	-1.650259	-0.473008	#Formamide
1	-6.860622	-0.675068	-0.462957	#Formamide
8	-3.878460	0.366030	-0.195095	#Formamide
6	1.799689	2.885190	-0.512680	#Formamide
1	1.481128	3.672137	-1.213449	#Formamide
7	2.716805	2.060173	-1.027101	#Formamide
1	3.088008	1.264126	-0.508173	#Formamide
1	3.035610	2.201338	-1.972386	#Formamide
8	1.330961	2.811749	0.622800	#Formamide

36
DAMN

6	-0.139154	1.376609	-1.962391	#DAMN
7	-0.221549	1.993992	-2.935750	#DAMN
6	-0.042248	0.682462	-0.726544	#DAMN
6	0.042248	-0.682462	-0.726544	#DAMN
6	0.139154	-1.376609	-1.962391	#DAMN
7	0.221549	-1.993992	-2.935750	#DAMN
7	0.118430	-1.443161	0.431908	#DAMN
7	-0.118430	1.443161	0.431908	#DAMN
1	-0.475851	-1.117740	1.202586	#DAMN
1	0.010323	-2.446507	0.272814	#DAMN
1	-0.010323	2.446507	0.272814	#DAMN
1	0.475851	1.117740	1.202586	#DAMN
6	0.179548	5.214509	-0.824289	#Formamide
1	0.287523	6.268174	-0.530359	#Formamide
7	0.118430	5.034996	-2.147643	#Formamide
1	0.016763	4.108382	-2.544861	#Formamide
1	0.170783	5.824797	-2.770501	#Formamide
8	0.124577	4.320952	0.019726	#Formamide
6	-0.179548	-5.214509	-0.824289	#Formamide
1	-0.287523	-6.268174	-0.530359	#Formamide
7	-0.118430	-5.034996	-2.147643	#Formamide
1	-0.016763	-4.108382	-2.544861	#Formamide
1	-0.170783	-5.824797	-2.770501	#Formamide
8	-0.124577	-4.320952	0.019726	#Formamide
6	2.637088	0.122983	2.682017	#Formamide
1	3.515711	0.295545	3.319817	#Formamide
7	2.695543	-1.004182	1.969286	#Formamide
1	1.913135	-1.268944	1.366627	#Formamide
1	3.493341	-1.614002	2.047289	#Formamide
8	1.702362	0.926571	2.655177	#Formamide
6	-2.637088	-0.122983	2.682017	#Formamide
1	-3.515711	-0.295545	3.319817	#Formamide
7	-2.695543	1.004182	1.969286	#Formamide
1	-1.913135	1.268944	1.366627	#Formamide
1	-3.493341	1.614002	2.047289	#Formamide
8	-1.702362	-0.926571	2.655177	#Formamide

References

1. Saladino, R.; Crestini, C.; Costanzo, G.; Negri, R.; Di Mauro, E. A possible prebiotic synthesis of purine, adenine, cytosine, and 4(3H)-pyrimidinone from formamide: Implications for the origin of life. *Bioorganic Med. Chem.* **2001**, *9*, 1249–1253.
2. Taniguchi, M.; Lindsey, J.S. Database of Absorption and Fluorescence Spectra of >300 Common Compounds for use in PhotochemCAD. *Photochem. Photobiol.* **2018**, *94*, 290–327.
3. Rimola, A.; Fabbiani, M.; Sodupe, M.; Ugliengo, P.; Martra, G. How Does Silica Catalyze the Amide Bond Formation under Dry Conditions? Role of Specific Surface Silanol Pairs. *ACS Catal.* **2018**, *8*, 4558–4568.
